# Cost-effectiveness analysis of NVX-CoV2373 COVID-19 vaccination for elderly people in Japan

**DOI:** 10.1016/j.jvacx.2024.100514

**Published:** 2024-06-20

**Authors:** Masafumi Kato, Takayori Ono, Hisato Deguchi, Norio Ohmagari, Ataru Igarashi

**Affiliations:** a Market Access, Public Affairs & Patient Experience, Japan Pharma Business Unit, Takeda Pharmaceutical Company Limited, Tokyo, Japan; b Medical Franchise Vaccine, Japan Medical Office, Takeda Pharmaceutical Company Limited, Tokyo, Japan; c Disease Control and Prevention Center, National Center for Global Health and Medicine Hospital, Tokyo, Japan; d AMR Clinical Reference Center, National Center for Global Health and Medicine Hospital, Tokyo, Japan; e Department of Public Health, School of Medicine, Yokohama City University, Yokohama, Japan

**Keywords:** COVID-19, Cost-effectiveness, Markov model, NVX-CoV2373, Vaccine

## Abstract

**Background:**

NVX-CoV2373 is one of the vaccines marketed for COVID-19 prevention in Japan. Information on its cost-effectiveness is important for making well-informed decisions on the future of Japan’s COVID-19 vaccination programme from the public healthcare payer’s perspective. The aim of this study was to evaluate the cost-effectiveness of NVX-CoV2373 vaccination in the elderly Japanese population.

**Methods:**

Two analysis populations that included elderly Japanese individuals (aged ≥ 65 years) were defined in this study: those who had not received a COVID-19 vaccine or had not completed a primary vaccination series (i.e., first two vaccinations) with an approved COVID-19 vaccine (analysis population 1), and those who had received two primary vaccinations with an approved COVID-19 vaccine (analysis population 2). A literature-informed Markov model for each analysis population was developed to evaluate the cost-effectiveness of vaccination with NVX-CoV2373 against no vaccination with NVX-CoV2373 from the public healthcare payer’s perspective as a base-case analysis and from the societal perspective as a scenario analysis. Vaccine efficacy was estimated from a phase 3 study of NVX-CoV2373 (EudraCT number: 2020–004123-16). Cost-effectiveness was assessed using a willingness-to-pay threshold of Japanese yen (JPY) 5 million per quality-adjusted life-year (QALY). Deterministic and probabilistic sensitivity analyses were also performed.

**Results:**

For analysis population 1, NVX-CoV2373 primary and booster vaccinations would reduce costs by JPY 37,647 and prolong QALYs by 0.01601. Therefore, NVX-CoV2373 primary and booster vaccinations were considered to be dominant over no vaccination. For analysis population 2, an NVX-CoV2373 booster vaccination would increase costs by JPY 5010 and prolong QALYs by 0.00550, with the incremental cost-effectiveness ratio of JPY 910,566 per QALY gained.

**Conclusions:**

Our analyses suggest that a vaccination strategy with NVX-CoV2373 is cost-effective in the elderly population (aged ≥ 65 years) of Japan*.*

## Highlights

• NVX-CoV2373 is a vaccine marketed for COVID-19 prevention in Japan.
• Its cost-effectiveness was assessed for the first time in elderly people in Japan.
• Primary and booster vaccinations were dominant over no vaccination.
• A booster vaccination cost more than no booster vaccination but prolonged QALYs.

## Introduction

Since the first cases of pneumonia of unknown aetiology were reported in December 2019 in Wuhan, China, there have been more than 770 million confirmed cases of coronavirus disease 2019 (COVID-19) and 6.96 million confirmed COVID-19-related deaths globally (as of 6 September 2023) [1]. Japan’s first case of COVID-19 was confirmed on 16 January 2020 and, as of 6 September 2023, Japan has had > 33.8 million confirmed cases of COVID-19 and 74,694 confirmed COVID-19-related deaths [1].

A mass vaccination programme, which deployed four different COVID-19 vaccines, was implemented in Japan under the Immunization Act [2], [3]. COVID-19 vaccines fell under the ‘temporary vaccination’ category of the Act, enabling free administration of the vaccines to Japan’s residents [2]. At the 45th Vaccination and Vaccine Subcommittee Meeting of the Health and Science Council of Japan, it was decided to continue free vaccinations against COVID-19 in the fiscal year of 2023 [4]. The elderly population and individuals with underlying health conditions, who are most at risk of serious COVID-19 complications, and healthcare professionals would have the opportunity to receive two COVID-19 vaccinations between May 2023 and December 2023, while all other individuals would receive one vaccination between September 2023 and December 2023 [4].

The ‘temporary vaccination’ program that has offered publicly funded COVID-19 vaccinations for all ages will finish at the end of the fiscal year of 2023. From the fiscal year of 2024, COVID-19 vaccinations will only be covered for the elderly population (i.e. individuals aged ≥ 65 years) and those aged 60 to 64 years with underlying medical conditions under a ‘routine vaccination’ program [5]. Given the enormous health and economic burden caused by COVID-19, information on the cost-effectiveness of the vaccinations is essential for making well-informed decisions on the future of the programme.

NVX-CoV2373 (Nuvaxovid) is a recombinant severe respiratory syndrome coronavirus 2 (SARS-CoV-2) spike protein nanoparticle vaccine with Matrix-M adjuvant that is manufactured by Novavax. NVX-CoV2373 received regulatory approval in Japan in April 2022 [6] and has been available in Japan’s COVID-19 vaccination programme since May 2022. To date, the cost-effectiveness of vaccination with NVX-CoV2373 in Japan has not been evaluated. The aim of this study was to assess the cost-effectiveness of NVX-CoV2373 vaccination in the elderly population (i.e., people aged ≥ 65 years), who are at high risk of severe COVID-19, from the public healthcare payer’s perspective in Japan. In individuals who had not received a COVID-19 vaccine or had not completed a primary vaccination series (i.e., first two vaccinations) with an approved COVID-19 vaccine, NVX-CoV2373 primary and booster vaccinations (i.e., three vaccinations) were compared with no vaccination. In individuals who had previously received two primary vaccinations with an approved COVID-19 vaccine, an NVX-CoV2373 booster vaccination (i.e., one vaccination) was compared with no booster vaccination.

## Materials and methods

A Markov state transition model was developed to estimate the cost-effectiveness of vaccination with NVX-CoV2373 against no vaccination in elderly individuals in Japan. The Markov model used a 1-day cycle, and results were reported over a 1-year time horizon. Discount rates were not accounted for owing to the short-term time horizon. The model was constructed and implemented using TreeAge Pro 2023 R1 and validated using a separate model with the same analysis conditions, which was constructed in Microsoft Excel.

### Analysis framework

Two different analysis populations were defined to evaluate the cost-effectiveness of NVX-CoV2373 vaccination in Japan, both of which consisted of elderly individuals aged ≥ 65 years (Table 1). Analysis population 1 comprised individuals who either had not received a COVID-19 vaccine or had not completed two primary vaccinations with an approved COVID-19 vaccine. Analysis population 2 comprised individuals who had received two primary vaccinations with an approved COVID-19 messenger RNA (mRNA) vaccine ≥ 180 days ago.

**Table 1:** Definitions of analysis populations.

**Analysis population**	**Definition**
Analysis population 1	Elderly Japanese individuals (aged ≥ 65 years) who had not received a COVID-19 vaccine OR Elderly Japanese individuals (aged ≥ 65 years) who had not completed two primary vaccinations with an approved COVID-19 vaccine
Analysis population 2	Elderly Japanese individuals (aged ≥ 65 years) who had received two primary vaccinations with an approved COVID-19 mRNA vaccine ≥ 180 days ago

COVID-19: coronavirus disease 2019.

For analysis population 1, NVX-CoV2373 primary and booster vaccinations (i.e., three vaccinations) were compared with no vaccination. For analysis population 2, an NVX-CoV2373 booster vaccination (i.e., one vaccination) was compared with no booster vaccination.

### Model structure

**Fig. 1:**
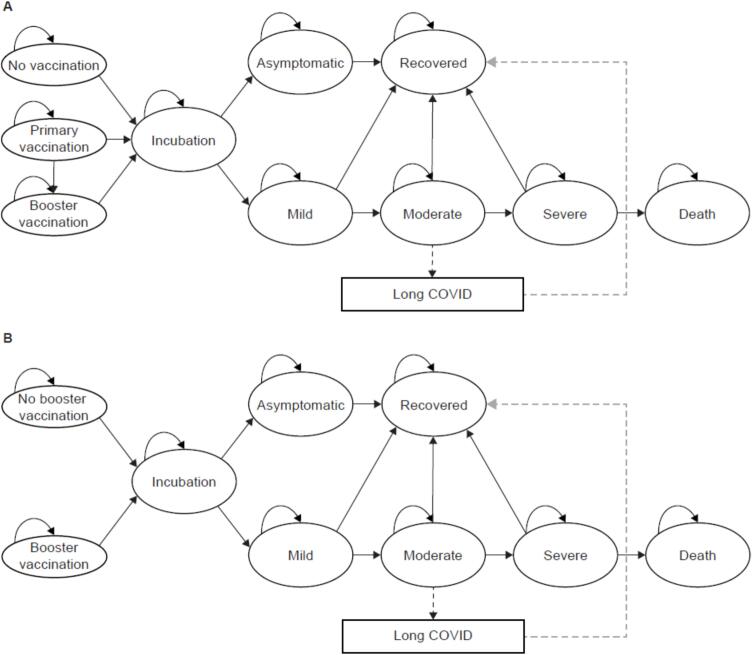
Model structure. (**A**) Analysis population 1 comprised Japanese elderly individuals aged ≥ 65 years who had either not received a COVID-19 vaccine or had not completed two primary vaccinations with an approved COVID-19 vaccine. (**B**) Analysis population 2 comprised Japanese elderly individuals aged ≥ 65 years who had received two primary vaccinations with an approved COVID-19 mRNA vaccine. COVID-19: coronavirus disease 2019; mRNA, messenger RNA.

The model structures were based on the structure used by Li et al. 2022 [7]. In analysis population 1, individuals in the ‘primary vaccination’ and ‘booster vaccination’ states both started from the ‘primary vaccination’ state, in which they received two NVX-CoV2373 vaccinations (Fig. 1**A**). They then received a third NVX-CoV2373 vaccination 180 days later and moved to the ‘booster vaccination’ state. Individuals in the comparator started from the ‘no vaccination’ state.

In analysis population 2, individuals who received an NVX-CoV2373 booster vaccination started from the ‘booster vaccination’ state, and individuals in the comparator started from the ‘no booster vaccination’ state (Fig. 1**B**).

The subsequent model structures were the same for analysis populations 1 and 2. With the exception of the ‘long COVID’ state, the structures were identical to those used by Li et al. 2022 [7]. If individuals were infected with SARS-CoV-2, they went through the ‘incubation’ state, followed by the states related to infection with symptoms or without symptoms (‘asymptomatic’). Symptomatic states were classified as ‘mild’, ‘moderate’, ‘severe’ or ‘death’. Mild, moderate and severe disease states were determined by how much intervention was needed: individuals with mild COVID-19 required outpatient treatment or hospitalization without supplemental oxygen; those with moderate COVID-19 required high- or low-flow supplemental oxygen; and those with severe COVID-19 required admission to the intensive care unit (ICU) or extracorporeal membrane oxygenation/invasive ventilation. Only individuals who had a symptomatic infection could develop long COVID.

### Model inputs

The model parameters used in the analysis are summarized in Table 2, Table 3.

**Table 2:** Model parameters.

**Item**				**Value**	**Settings in DSA**			**Settings in PSA**			**Source**
					**Lower limit**	**Upper limit**	**Range**	**Distribution**	**Parameters**^a^		
**VE of NVX-CoV2373 vaccine (%)**											

Analysis population 1	Against infection	NVX-CoV2373 primary vaccinations		88.9	20.2	99.7	95% CI	Beta (SE 10%)	10.21	1.27	[8] (aged ≥ 65 years)

	NVX-CoV2373 booster vaccination		88.9	20.2	99.7	95% CI	Beta (SE 10%)	10.21	1.27		

Against severe disease	NVX-CoV2373 primary vaccinations		86.9	73.7	93.5	95% CI	Beta (SE 10%)	12.23	1.84		

NVX-CoV2373 booster vaccination		86.9	73.7	93.5	95% CI	Beta (SE 10%)	12.23	1.84

Analysis population 2	Against infection	NVX-CoV2373 booster vaccination		88.9	20.2	99.7	95% CI	Beta (SE 10%)	10.21	1.27

Against severe disease	NVX-CoV2373 booster vaccination		86.9	73.7	93.5	95% CI	Beta (SE 10%)	12.23	1.84

**VE of COVID-19 mRNA vaccine (%)**											

Analysis population 2	Against infection	No booster vaccination^b^		50.6	14.4	71.5	95% CI	Beta (SE 10%)	48.89	47.73	[9]

Against severe disease	No booster vaccination^b^		83.4	66.7	100.0	±20%	Beta (SE 10%)	15.77	3.14	[10], [11]

**Incidence of COVID-19 (per 100,000 person-days)**											

Analysis population 1	No vaccination			71.5	57.2	85.8	±20%	Triangular^c^	Min: 13.53 Max: 175.71	Mode: 71.53	[9], [12], [14], [28]

NVX-CoV2373 primary and booster vaccinations^d^			7.9	-	-	-	-	-	-	[28], [8] (aged ≥ 65 years)

Analysis population 2	No booster vaccination^d^			35.3	-	-	-	-	-	-	[9], [28]

NVX-CoV2373 booster vaccination^d^			7.9	-	-	-	-	-	-	[28], [8] (aged ≥ 65 years)

**Distribution of clinical outcomes (%)**											

No vaccination	Asymptomatic infection			35.6	-	-	-	Dirichlet	0.36	-	[16]

	Mild disease			49.5	-	-	-		0.50	-	-

	Moderate disease			9.4	-	-	-		0.09	-	[17]

Severe disease			1.1	-	-	-	0.01	-	[18]

Death			4.4	-	-	-	0.04	-	[18]

**Duration of clinical stages (days)**											

Incubation period				2.9	2.6	3.2	95% CI	Gamma	358.98	0.01	[15]

Asymptomatic infection				6.0	4.8	7.2	±20%	Gamma (SE 10%)	100	0.06	[7]

Mild disease	In mild state			11.0	10.9	11.1	95% CI	Gamma	21,040.90	0.001	Data are averages calculated using the MDV claims database

Moderate disease	In mild state			11.2	10.9	11.5	95% CI	Gamma	6970.60	0.002	

In moderate state			6.4	6.3	6.5	95% CI	Gamma	7190.70	0.001		

Severe disease	In mild state			6.9	6.3	7.5	95% CI	Gamma	591.33	0.012

	In moderate state			2.6	2.2	3.0	95% CI	Gamma	183.43	0.014

In severe state			8.6	8.3	8.9	95% CI	Gamma	3886.45	0.002

In recovery state			5.7	4.6	6.8	±20%	Gamma (SE 10%)	100	0.06	[7]

Death^e^	In mild state			3.4	2.7	4.1	95% CI	Gamma	85.27	0.040	Data are averages calculated using the MDV claims database

	In moderate state			4.9	4.1	5.7	95% CI	Gamma	162.41	0.030	

In severe state			8.7	7.8	9.6	95% CI	Gamma	391.32	0.022

**Cumulative incidence of long COVID (%)**				40.1	34.7	45.5	95% CI	Beta	126.75	189.34	[19]

**Duration of long COVID (days)**				760.2 (108.6 weeks)	608.2	912.2	±20%	Log-normal (SE 10%)	6.63	0.01	[20]

**Costs (JPY)**											

Vaccination cost	NVX-CoV2373 vaccine price			9000	4500	13,500	±50%	Gamma (SE 10%)	100	90.00	Assumption for the analysis

Vaccine administration			3700	2960	4440	±20%	Gamma (SE 10%)	100	37.00	[22]

Treatment costs for COVID-19^f^^,^^g^	Recovered	Asymptomatic infection		0	-	-	-	-	-	-	

		Mild disease (JPY 93,242)	Outpatient expenses^h^	8035	6428	9642	±20%	Gamma (SE 10%)	100	80.35	

			Hospitalization expenses	804,361	795,547	813,175	95% CI	Gamma	31,992.95	25.14	

	Hospitalization rate (%)	10.7	8.6	12.8	±20%	Beta (SE 10%)	89.19	744.39	[43]

Moderate disease		1,515,231	1,498,133	1,532,329	95% CI	Gamma	30,168.88	50.22	

Severe disease		3,085,669	3,006,771	3,164,567	95% CI	Gamma	5875.90	525.14	

Death	Severe disease		3,320,958	3,037,040	3,604,876	95% CI	Gamma	525.60	6318.44	

Treatment cost for long COVID				17,640	14,112	21,168	±20%	Gamma (SE 10%)	100	176.40	

**Utilities**											

Utility of COVID-19	Asymptomatic infection			0.8695	0.6956	1.0000	±20%	Beta (SE 10%)	12.18	1.83	[24]

	Mild disease			0.5095	0.4076	0.6114	±20%	Beta (SE 10%)	48.54	46.73	[20]

	Moderate disease			0.2895	0.2316	0.3474	±20%	Beta (SE 10%)	70.76	173.66	

Severe disease			0.0000	-	-	-	-	-	-

Recovered			0.5095	0.4076	0.6114	±20%	Beta (SE 10%)	48.54	46.73	Assumption

Disutility of long COVID				−0.1100	−0.1300	−0.0900	95% CI	Beta	103.32	835.92	[25]

CI: confidence interval; COVID-19: coronavirus disease 2019; DSA: deterministic sensitivity analysis; ICU: intensive care unit; JPY: Japanese yen; MDV: Medical Data Vision Company Limited; mRNA: messenger RNA; PSA: probabilistic sensitivity analysis; SE: standard error; VE: vaccine efficacy.

a Beta (α, β), triangular (min, max, mode), Dirichlet (α), gamma (α, β), log-normal (μ, σ).

b VE of COVID-19 mRNA vaccine 180 days after completing the second primary vaccination (individuals who had only received one vaccination were considered to have no immunity and, therefore, no VE).

c The minimum and maximum values during the evaluation period (July 2022 to October 2022) were set as parameters.

d Values were calculated using the equation: infection rate = Y × (1 − VE % against infection), with Y being the infection rate in unvaccinated individuals (i.e., 71.5 per 100,000 person-days). Ranges for the sensitivity analyses were calculated using the ranges provided for Y and the respective VEs.

e The duration of the clinical stages was assumed to be the same as for severe disease.

f Treatment costs from the onset of COVID-19 to recovery or death.

g Treatment costs were calculated by multiplying hospitalization charges by the number of days of the clinical stage. For moderate and severe disease, the cost of supplemental oxygen inhalation and of ICU admission and ventilation, respectively, were calculated during hospitalization. It was assumed that 10.7 % of patients with mild cases would be hospitalized [23]. Outpatient visits and antigen test costs were calculated once for individuals who were not hospitalized. For the treatment of long COVID, it was assumed that individuals had one outpatient visit (consultation fee was JPY 735) per month for the duration of long COVID.

h Outpatient expenses were the sum of the consultation fee (JPY 735), the in-hospital triage fee (JPY 3000) and the antigen test cost (JPY 4300).

**Table 3:** Model parameters for the scenario analyses.

**Item**		**Value**	**Source**
**Scenario 1: incidence of COVID-19 (per 100,000 person-days) based on data from the MHLW’s HER-SYS**			
Analysis population 1	No vaccination	33.1	[28]
	NVX-CoV2373 primary and booster vaccinations^a^	3.7	[28], [8] (aged ≥ 65 years)
Analysis population 2	No booster vaccination^a^	16.3	[9], [28]
	NVX-CoV2373 booster vaccination^a^	3.7	[28], [8] (aged ≥ 65 years)

**Scenario 2: VE (%) of COVID-19 mRNA vaccine (from two primary vaccinations) against infection was based on data obtained during the omicron epidemic in Japan**			
Analysis population 2	No booster vaccination^b^	35.0	[29]
	NVX-CoV2373 booster vaccination	88.9	[8] (aged ≥ 65 years)

**Scenario 3: incorporation of productivity loss (JPY/day)**			
Productivity loss	Absence from work due to COVID-19 vaccination	0.5 days	Assumption
	Productivity loss due to COVID-19^c^	2280	[30], [31]

**Scenario 4: VE (%) of NVX-CoV2373 against infection based on data from all ages (i.e. 18–84 years) in the 2019nCoV-302 study**			
Analysis population 1	NVX-CoV2373 primary vaccinations	89.7	[8] (aged 18–84 years)
	NVX-CoV2373 booster vaccination	89.7	
Analysis population 2	NVX-CoV2373 booster vaccination	89.7	

COVID-19: coronavirus disease 2019; HER-SYS: Health Center Real-time Information-sharing System on COVID-19; JPY: Japanese yen; MHLW: Ministry of Health, Labour and Welfare; mRNA: messenger RNA; VE: vaccine efficacy.

a Values were calculated using the equation: infection rate = Y × (1 − VE % against infection), with Y being the infection rate in unvaccinated individuals (i.e., 71.5 per 100,000 person-days). Ranges for the sensitivity analyses were calculated using the ranges provided for Y and the respective VEs.

b VE of COVID-19 mRNA vaccine 180 days after completing the second primary vaccination (individuals who had only received one vaccination were considered to have no immunity and, therefore, no VE).

c Calculated using the employment rate of adults aged ≥ 65 years in Japan (25.4 %), the proportion of regular employees (47.8 %) and non-regular employees (44.7 %), and the average wage in the target population (regular employees: JPY 11,222 per day; non-regular employees: JPY 8074 per day).

#### Vaccine efficacy

The vaccine efficacy (VE) of NVX-CoV2373 against infection and severe disease was 88.9 % and 86.9 %, respectively; these values were based on data from elderly individuals (aged ≥ 65 years) in the pivotal phase 3 study of NVX-CoV2373 in the UK (the 2019nCoV-302 study) [8]. The 2019nCoV-302 study took place during the period in which the SARS-CoV-2 alpha variant was becoming more prevalent. We have assumed that VE will be similar for omicron variants owing to lack of evidence of VE against omicron variants. It was also assumed that the VE of primary vaccinations was maintained by a booster vaccination and that VE did not wane over the 1-year time horizon.

Among individuals in analysis population 2, VE of COVID-19 mRNA vaccines from their primary vaccinations against infection was 50.6 %; this was based on data derived from the period in which the delta variant was prevalent in Japan [9]. VE against severe disease was 83.4 %; this was based on studies conducted in Israel and Qatar in 2021 [10], [11].

#### COVID-19 infection rates

Similar to the method devised by Li et al. 2022 [7], the calculation of COVID-19 infection rates in unvaccinated individuals (i.e., individuals in analysis population 1 who did not receive NVX-CoV2373 primary and booster vaccinations) used relevant data derived from the period in which the omicron BA.5 variant was dominant. The average number of COVID-19 cases was calculated to be 34.5 cases per 100,000 person-days using data reported by the Ministry of Health, Labour and Welfare (MHLW) between 29 June 2022 and 1 November 2022 in individuals aged ≥ 60 years [12], [13]. Although current case numbers of COVID-19 (i.e. as of September 2023) in Japan are not available, it is possible that the incidence of COVID-19 is currently higher than it was between 29 June 2022 and 1 November 2022. Other variables were based on the following: the percentage of unvaccinated individuals aged ≥ 60 years (defined as those who had not had a COVID-19 vaccination or had not received two primary vaccinations with a COVID-19 vaccine); the percentage of individuals aged ≥ 60 years who had received one to four vaccinations with a COVID-19 vaccine during the same period [14]; and the VE of two to four vaccinations with a COVID-19 mRNA vaccine in individuals aged ≥ 65 years as reported in the VERSUS study [9].

The infection rate of individuals in analysis population 1 who did not receive NVX-CoV2373 primary and booster vaccinations (71.5 cases per 100,000 person-days) was used to calculate the COVID-19 infection rates in individuals in analysis population 1 who received NVX-CoV2373 primary and booster vaccinations (7.9 cases per 100,000 person-days), individuals in analysis population 2 who received an NVX-CoV2373 booster vaccination (7.9 cases per 100,000 person-days) and individuals in the ‘no booster vaccination’ comparator of analysis population 2 (35.3 cases per 100,000 person-days).

#### Distribution of clinical outcomes and duration of conditions

An incubation period of 2.9 days was used. This was the median incubation period for the omicron B.1.1.529 variant reported by the National Institute of Infectious Diseases [15]. Individuals with COVID-19 could be asymptomatic, have mild, moderate or severe disease, or die. The rates of these outcomes in unvaccinated individuals were estimated using data from publicly available sources in Japan [16], [17], [18]. It was assumed that 64.4 % of unvaccinated individuals with COVID-19 would develop symptoms: 49.5 % of individuals would have mild disease; 9.4 % would have moderate disease; 1.1 % would have severe disease; and 4.4 % would die. The proportions of clinical outcomes in vaccinated individuals were calculated based on the model developed by Li et al. 2022 [7], the rates of clinical outcomes in unvaccinated individuals (five outcomes were converted into two outcomes: ‘non-severe disease’ included asymptomatic and mild cases; and ‘severe disease’ included moderate, severe and fatal cases) and the appropriate VEs shown in Table 2. It was assumed that the proportions of asymptomatic and mild cases, and moderate, severe, and fatal cases in vaccinated individuals were equal to those in unvaccinated individuals. Of those vaccinated or unvaccinated individuals who develop symptomatic COVID-19, 40.1 % would develop long COVID; this was the cumulative incidence of long COVID reported by Fukunaga et al. 2023 [19] 3 months after a COVID-19 diagnosis.

It was assumed that individuals with asymptomatic infection were no longer infectious after 6 days [7]. The number of days that individuals took to reach the ‘recovery’ state when they had mild, moderate or severe disease was calculated by dividing the average treatment costs by the total number of hospitalization days, as outlined in the next ‘Costs’ section. The calculated number of days were 11.0 days for mild disease, 17.6 days for moderate disease (11.2 days in the ‘mild’ state and 6.4 days in the ‘moderate’ state) and 18.1 days for severe disease (6.9 days in the ‘mild’ state, 2.6 days in the ‘moderate’ state and 8.6 days in the ‘severe’ state).

The durations of the ‘mild’ and ‘moderate’ states for moderate disease and of the ‘mild’, ‘moderate’ and ‘severe’ states for severe disease were estimated using the median duration from onset of symptoms to the need for supplemental oxygen (mild state) and the median duration from the start of supplemental oxygen to ICU admission (moderate state). An individual with severe disease was further medically treated for 5.7 days before reaching the ‘recovery’ state [7]. For individuals who died, the duration of each state was assumed to be the same as the durations for severe disease. The duration of long COVID (108.6 weeks) was based on the length of time used by the National Institute for Health and Care Excellence (NICE) [20]. Transition probabilities (*p*) between health states were estimated using the following equation, with ‘*r*’ denoting the daily transition rate [7], [21]: *p* + 1 − exp^−^*^r^.*

#### Costs

The overall cost of an NVX-CoV2373 vaccination included the cost of the vaccine (Japanese yen [JPY] 9000 per vaccine [assumption for the analysis]) and vaccine administration (JPY 3700 per vaccination [22]). Average treatment costs for COVID-19 were calculated using the Medical Data Vision Company Limited claims database in Japan (hospitalized patients: JPY 804,361; outpatients: JPY 8035). Depending on disease severity (except for death), the costs were calculated as weighted mean percentages of hospitalized patients and outpatients. It was assumed that 10.7 % of people would be hospitalized [23]. The treatment cost for long COVID (JPY 17,640) was based on average monthly outpatient consultation fees (JPY 735 per visit for 108.6 weeks).

#### Utilities

The utility value for the asymptomatic infection state was assumed to be the same as the value for the general population in Japan aged ≥ 60 years (i.e., 0.8695) [24]. Utility values for mild to severe disease states were derived by subtracting the NICE utility decrements (i.e., values set in the UK) [20] from the health-related quality of life of the general population in Japan (mild disease: 0.5095; moderate disease: 0.2895; severe disease: 0.0000) [24]. The utility value for the recovered state was assumed to be the same as the value for the mild disease state. The disutility value of long COVID (−0.1100) was based on the EQ-5D-3L score determined in a survey of Japanese participants aged ≥ 20 years who had a documented history of COVID-19 [25].

### Base-case analysis

The model estimated the cost-effectiveness of the vaccination strategies for analysis populations 1 and 2 over a 1-year time horizon that commenced after the primary vaccinations for analysis population 1 and after the booster vaccination for analysis population 2. Quality-adjusted life-years (QALYs) and the incremental cost-effectiveness ratio (ICER) were used to assess the cost-effectiveness of the abovementioned vaccination strategies against no vaccination and were calculated for both analysis populations. A willingness-to-pay (WTP) threshold of JPY 5 million per QALY was used to assess cost-effectiveness, and the vaccinations were considered cost-effective if the estimated ratio was lower than this threshold [26], [27]. All costs included in the model are expressed in JPY in 2022.

### Sensitivity analyses

To test parameter uncertainty and assess the robustness of the results, deterministic sensitivity analyses (DSAs) and probabilistic sensitivity analyses (PSAs) were performed with the settings shown in Table 2. For DSAs, the lower and upper limits of the two-sided confidence intervals (CIs) were used as the lower and upper limits of the analyses, respectively. Parameters for which CI information was not available were set within ± 20 % of the value. The cost of the NVX-CoV2373 vaccine (JPY 9000 per dose) was set within the range of ± 50 %.

For PSAs, simulation was performed by generating random numbers according to a predetermined probability distribution for each variable and by repeating data extraction 10,000 times. If a standard error (SE) was not reported or could not be estimated for a parameter, the SE was assumed to be 10 % of the set value.

### Scenario analyses

Cost-effectiveness of the vaccination strategies in analysis populations 1 and 2 were also analysed under the following four scenarios, which used the parameters displayed in Table 3.

In scenario 1, information about new COVID-19 cases and vaccination coverage was based on data from the MHLW’s Health Center Real-time Information-sharing System (HER-SYS) [28]. There has been some concern about the accuracy and completeness of these records, which may be reflected in the lower infection rates than those used for the base-case analysis.

In scenario 2, VE of a COVID-19 mRNA vaccine (from two primary vaccinations) against infection was lower than the value used for the base-case analysis (i.e., 35.0 % vs. 50.6 % for the base-case analysis) and based on data obtained during the omicron epidemic in Japan rather than during the period when the delta variant was prevalent [29].

In scenario 3, which analysed cost-effectiveness from a societal perspective, absence from work and loss of productivity associated with COVID-19 were considered. Productivity loss was calculated using the employment rate of adults aged ≥ 65 years in Japan (25.4 %), the proportion of regular employees (47.8 %) and non-regular employees (44.7 %), and the average wage in the target population (regular employees: JPY 11,222 per day; non-regular employees: JPY 8074 per day) [30], [31].

In scenario 4, VE of NVX-CoV2373 against infection was slightly higher than the value used for the base-case analysis (i.e., 89.7 % vs. 88.9 % for the base-case analysis) and based on data from all ages (18–84 years) rather than only from adults aged ≥ 65 years in the 2019nCoV-302 study [8].

## Results

### Base-case analysis

Over the 1-year time horizon, NVX-CoV2373 primary and booster vaccinations in analysis population 1 were estimated to cost JPY 50,480 per person and lead to a gain of 0.867 QALYs. In contrast, no vaccination was estimated to cost JPY 88,127 per person and lead to a gain of 0.851 QALYs (Table 4). NVX-CoV2373 primary and booster vaccinations were dominant against the no vaccination strategy. In analysis population 2, an NVX-CoV2373 booster vaccination was estimated to cost JPY 25,261 per person and lead to a gain of 0.867 QALYs (Table 4). In contrast, no booster vaccination was estimated to cost JPY 20,251 per person and lead to a gain of 0.862 QALYs. The resulting ICER of a booster vaccination compared with no booster vaccination was JPY 910,566 per QALY.

**Table 4:** Base-case analysis.

	**Strategy**	**Cost (JPY)**	**Incremental cost (JPY)**	**Effectiveness (QALY)**	**Incremental effectiveness (QALY)**	**ICER (JPY/QALY)**
Analysis population 1	Primary and booster vaccinations	50,480	−37,647	0.86714	0.01601	Dominant
	No vaccination	88,127	-	0.85113	-	-
Analysis population 2	Booster vaccination	25,261	5010	0.86714	0.00550	910,566
	No booster vaccination	20,251	-	0.86163	-	-

ICER: incremental cost-effectiveness ratio; JPY: Japanese yen; QALY: quality-adjusted life-year.

### Deterministic sensitivity analyses

**Fig. 2:**
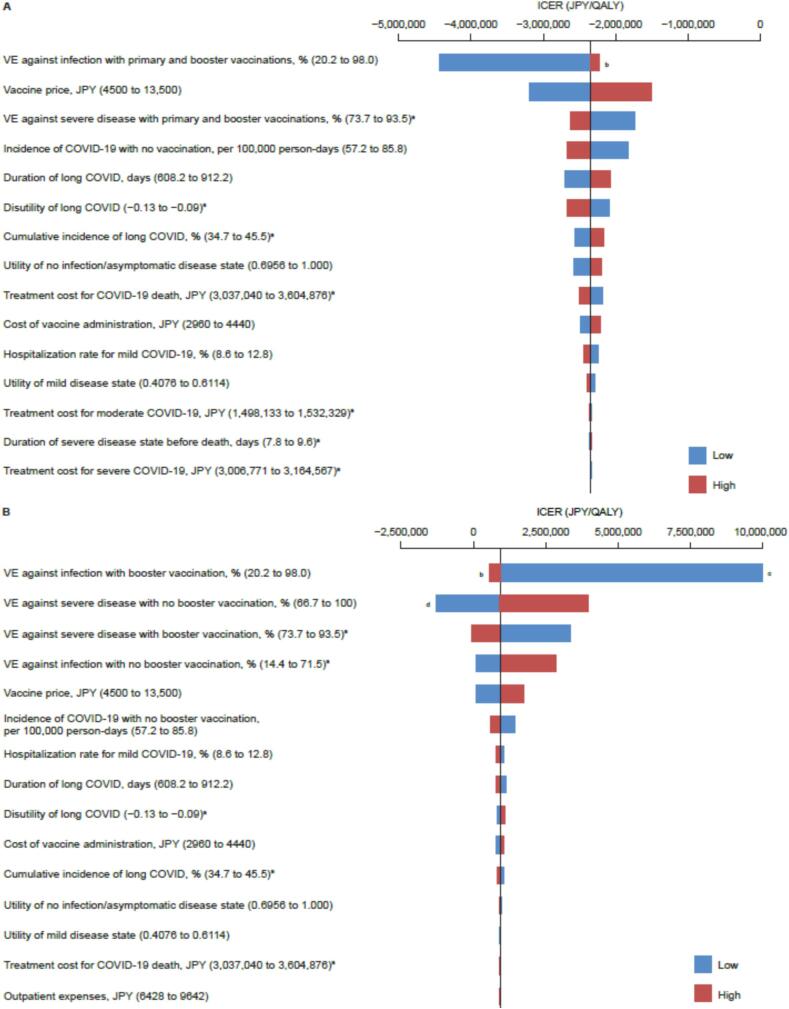
Tornado diagrams of deterministic sensitivity analyses showing the 15 variables with the largest impact on the ICER of (**A**) NVX-CoV2373 primary and booster vaccinations compared with no vaccination (analysis population 1) and (**B**) NVX-CoV2373 booster vaccination compared with no booster vaccination (analysis population 2). Parameters for which CIs were not available were set within ± 20 % of the value. The price of the NVX-CoV2373 vaccine (JPY 9000 per vaccination) was set within the range of ± 50 %. The vertical lines represent the ICERs for the base-case scenarios. A horizontal bar was generated for each parameter. The width of the bars indicates the potential effect of the parameters on the ICER when parameters are changed within their ranges. The red sections of each bar show high values of parameter ranges, and the blue sections show low values. ^a^ 95 % CI was used as the range for the sensitivity analysis. ^b^ Given that the distribution of clinical outcomes is not calculated correctly when the analysis is performed with an upper limit of 99.7 %, the analysis was performed with the upper limit set to 98.0 %, a point at which a reasonable distribution of clinical outcomes is obtained. ^c^ The ICER was JPY 5 million or greater when the value for VE against infection was ≤ 61 %, and it became dominant when the value was ≤ 49 %. ^d^ The ICER became dominant when the value for VE against severe disease was ≤ 77 %. CI: confidence interval; COVID-19: coronavirus disease 2019; ICER: incremental cost-effectiveness ratio; JPY: Japanese yen; QALY: quality-adjusted life-year; VE: vaccine efficacy. (For interpretation of the references to colour in this figure legend, the reader is referred to the web version of this article.)

DSAs showed that varying any individual model parameter did not alter the finding that NVX-CoV2373 primary and booster vaccinations were dominant against the no vaccination strategy in analysis population 1. The results suggest that the ICER was most sensitive to the parameter ‘VE against infection with primary and booster vaccinations’ (Fig. 2**A**), followed by the parameter ‘NVX-CoV2373 vaccine price’.

Within the ranges selected for the DSAs, the cost-effectiveness of NVX-CoV2373 booster vaccination compared with no booster vaccination remained unchanged in analysis population 2. The results suggest that the ICER of an NVX-CoV2373 booster vaccination compared with no booster vaccination was most sensitive to the parameter ‘VE against infection with booster vaccination’ (Fig. 2**B**), followed by the parameter ‘VE against severe disease with booster vaccination’.

### Probabilistic sensitivity analyses

**Fig. 3:**
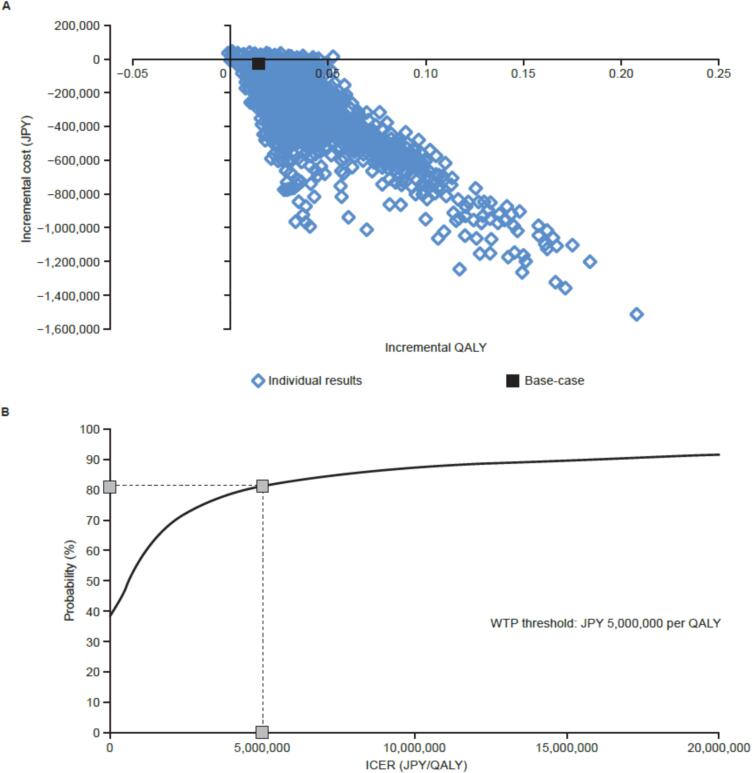
Probabilistic sensitivity analysis of NVX-CoV2373 primary and booster vaccinations compared with no vaccination in analysis population 1. (**A**) Scatter plot of incremental costs and QALYs. (**B**) Cost-effectiveness acceptability curve. At a threshold of JPY 5 million per QALY, NVX-CoV2373 primary and booster vaccinations had a probability of 81.5 % for being cost-effective. JPY: Japanese yen; QALY: quality-adjusted life-year; WTP: willingness-to-pay.

**Fig. 4:**
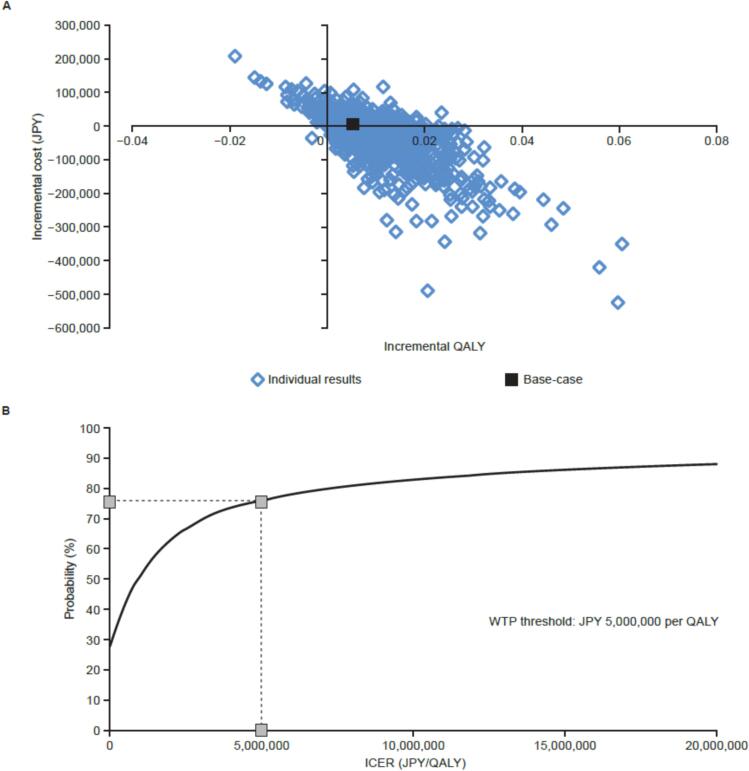
Probabilistic sensitivity analysis of an NVX-CoV2373 booster vaccination compared with no booster vaccination in analysis population 2. (**A**) Scatter plot of incremental costs and QALYs. (**B**) Cost-effectiveness acceptability curve. At a threshold of JPY 5 million per QALY, an NVX-CoV2373 booster vaccination had a probability of 75.9 % for being cost-effective. JPY: Japanese yen; QALY: quality-adjusted life-year; WTP: willingness-to-pay.

Of the 10,000 simulations performed in the PSAs, the ICERs for the vaccination strategies in analysis populations 1 and 2 mostly remained in the southeast quadrant (Fig. 3**A,** Fig. 4**A**), indicating that the vaccination strategies improved health and were less costly than no vaccination. When the WTP threshold of the ICER was set to JPY 5 million per QALY gained, the cost-effectiveness acceptability curve estimated an 81.5 % probability that NVX-CoV2373 primary and booster vaccinations would be cost-effective compared with no vaccination (Fig. 3**B**). The probability that an NVX-CoV2373 booster vaccination would be cost-effective compared with no booster vaccination was estimated to be 75.9 % (Fig. 4**B**).

### Scenario analyses

Four different scenarios were analysed to assess their effect on the cost-effectiveness of the NVX-CoV2373 vaccination strategies in analysis populations 1 and 2 (Table 5).

**Table 5:** Scenario analyses.

	**Strategy**	**Cost (JPY)**	**Incremental cost (JPY)**	**Effectiveness (QALY)**	**Incremental effectiveness (QALY)**	**ICER (JPY/QALY)**
**Scenario 1: incidence of COVID-19 based on data from the MHLW’s HER-SYS**						
Analysis population 1	Primary and booster vaccinations	43,866	393	0.86840	0.00792	49,667
	No vaccination	43,473	-	0.86048	-	-
Analysis population 2	Booster vaccination	18,550	8878	0.86840	0.00265	3,345,909
	No booster vaccination	9672	-	0.86575	-	-

**Scenario 2: VE (%) of COVID-19 mRNA vaccine (from two primary vaccinations) against infection was based on data obtained during the omicron epidemic in Japan**						
Analysis population 1^a^	Primary and booster vaccinations	-	-	-	-	-
	No vaccination	-	-	-	-	-
Analysis population 2	Booster vaccination	25,261	3197	0.86714	0.00752	424,895
	No booster vaccination	22,064	-	0.85961	-	-

**Scenario 3: incorporation of productivity loss**						
Analysis population 1	Primary and booster vaccinations	54,437	−37,998	0.86714	0.01601	Dominant
	No vaccination	92,435	-	0.85113	-	-
Analysis population 2	Booster vaccination	26,955	4764	0.86714	0.00550	865,886
	No booster dose	22,190	-	0.86163	-	-

**Scenario 4: VE of NVX-CoV2373 against infection based on data from all ages (i.e., 18–84 years) in the 2019nCoV-302 study**						
Analysis population 1	Primary and booster vaccinations	50,385	−37,742	0.86725	0.01613	Dominant
	No vaccination	88,127	-	0.85113	-	-
Analysis population 2	Booster vaccination	25,153	4902	0.86725	0.00562	872,470
	No booster vaccination	20,251	-	0.86163	-	-

HER-SYS: Health Center Real-time Information-sharing System; ICER: incremental cost-effectiveness ratio; JPY: Japanese yen; MHLW: Ministry of Health, Labour and Welfare; mRNA: messenger RNA; QALY: quality-adjusted life-year; VE: vaccine efficacy.

a This scenario is not applicable for analysis population 1.

In scenario 1, when the incidence of COVID-19 was lower than the rates used for the base-case analysis (i.e., based on data from the MHLW’s HER-SYS), the ICER of NVX-CoV2373 primary and booster vaccinations compared with no vaccination in analysis population 1 was JPY 49,667 per QALY. The ICER of an NVX-CoV2373 booster vaccination compared with no booster vaccination in analysis population 2 was JPY 3,345,909 per QALY.

In scenario 2, when the VE of a COVID-19 mRNA vaccine (from two primary vaccinations) against infection was lower than the value used for the base-case analysis (i.e., 35.0 % vs. 50.6 %; based on data obtained during the omicron epidemic in Japan), the ICER of an NVX-CoV2373 booster vaccination compared with no booster vaccination in analysis population 2 was JPY 424,895 per QALY.

In scenario 3, when the model was adjusted for productivity loss, NVX-CoV2373 primary and booster vaccinations were dominant against the no vaccination strategy in analysis population 1. The ICER of an NVX-CoV2373 booster vaccination compared with no booster vaccination in analysis population 2 was JPY 865,886 per QALY.

In scenario 4, when the VE of NVX-CoV2373 against infection was based on data from all ages (18–84 years) in the 2019nCoV-302 study, NVX-CoV2373 primary and booster vaccinations were dominant against the no vaccination strategy in analysis population 1. The ICER of an NVX-CoV2373 booster vaccination compared with no booster vaccination in analysis population 2 was JPY 872,470 per QALY.

In all analysed scenarios, NVX-CoV2373 primary and booster vaccinations (analysis population 1) or an NVX-CoV2373 booster vaccination only (analysis population 2) were cost-effective compared with no vaccination.

## Discussion

This study is the first cost-effectiveness analysis of NVX-CoV2373 vaccination in elderly individuals (aged ≥ 65 years) conducted in Japan. VE of NVX-CoV2373 was based on data from the pivotal phase 3 2019nCoV-302 study [8]. Overall, in analysis population 1, NVX-CoV2373 primary and booster vaccinations over the 1-year time horizon were dominant against the no vaccination strategy (i.e., improved health outcomes and reduced cost). In analysis population 2, the ICER of an NVX-CoV2373 booster vaccination compared with no booster vaccination over the 1-year time horizon was JPY 910,556 per QALY. These results indicate that both vaccination strategies are highly likely to be cost-effective in the target population, with a WTP threshold of JPY 5 million per QALY of the ICER.

With the threshold set at JPY 5 million per QALY, the probability that the vaccination strategies would be cost-effective compared with no vaccination was estimated to be 81.5 % and 75.9 % in analysis populations 1 and 2, respectively. DSAs suggest that the ICERs were most sensitive to the parameters ‘VE against infection with primary and booster vaccinations’ and ‘VE against infection with booster vaccination’.

The results of the scenario analyses were similar to those of the base-case analysis and demonstrated that the following factors do not affect the conclusion that NVX-CoV2373 primary and booster vaccinations (analysis population 1) or an NVX-CoV2373 booster vaccination only (analysis population 2) are likely to be cost-effective compared with no vaccination: the impact of lower infection rates (data from the MHLW’s HER-SYS rather than from the Vaccination Record System and the VERSUS study); lower VE of a COVID-19 mRNA vaccine (from two primary vaccinations) against infection (determined during the omicron epidemic in Japan rather than a time when the delta variant was prevalent); loss of productivity in the vaccinated working population; and slightly higher VE of NVX-CoV2373 against infection (data from adults of all ages rather than adults aged ≥ 65 years in the 2019nCoV-302 study).

The COVID-19 pandemic has caused overwhelming strain on economies and healthcare systems around the world. Even after 3 years of the pandemic, there is still a shortage of hospital beds during spikes in infections [32]. In Japan, the economic costs of the pandemic are forecasted to be at least JPY 194.7 trillion by 2030 [33]. COVID-19 vaccinations are considered to be the most critical strategy to control COVID-19, and governments worldwide implemented mass vaccination programmes to ensure sufficient hospital capacity during the pandemic. Health economic evaluations of COVID-19 vaccinations, including cost-effectiveness analyses, are essential for making well-informed decisions on the future of these vaccination programmes. This study represents an initial attempt to understand the cost-effectiveness of NVX-CoV2373 vaccinations in elderly individuals in Japan. As of September 2023, 69 % of the total population and 92 % of elderly individuals (aged ≥ 65 years) in Japan have received COVID-19 primary and booster vaccinations [34]. Considering the rapid change to the vaccination status of Japan’s residents due to the mass vaccination programme, a cost-effectiveness analysis of an NVX-CoV2373 booster vaccination in a population who has received primary and booster vaccinations would be beneficial for the current situation in Japan.

This study has several limitations. First, long-term VE data and data demonstrating waning of immunity from natural infection were not available at the time of this analysis. Thus, we were unable to incorporate any assumption in the model regarding attenuation of vaccine protection during the analysis period, which may have resulted in an overestimation of VE. Second, at the time of this analysis, data on VE of NVX-CoV2373 were only available for primary vaccinations against SARS-CoV-2 variants of the time. It has been recently reported that, following NVX-CoV2373 vaccinations, anti-SARS-CoV-2 immune responses against omicron variants are of a lower magnitude than those against the ancestral strain [35], [36]. However, NVX-CoV2373 booster vaccinations resulted in enhanced cross-reactive immunity to SARS-CoV-2 variants, with the magnitude of difference in immune responses between the ancestral strain and omicron variants diminishing with each booster vaccination [35]. Therefore, the VE of NVX-CoV2373 against current epidemic strains may be lower than the VE against the ancestral strain. We cannot exclude the possibility that the VE of a primary vaccination may not be the same as that of a booster vaccination; however, immunogenicity data from studies of NVX-CoV2373 booster vaccinations [35], [37] suggest that the magnitude of difference in VE are not critical. A third limitation of the study is that the utility values were based on values set in the UK [20], with the exception of the durations of asymptomatic infection and long COVID.

Several cost-effectiveness analyses of primary and booster vaccinations with various COVID-19 vaccine products conducted in Brazil, Hong Kong, Poland and the US have been reported, with many concluding that mass vaccination strategies are likely to be cost-saving, in particular in elderly populations [7], [38], [39], [40], [41], [42]. However, caution is required when extrapolating study findings, including extrapolating those from our study to other settings, because these analyses are all subject to limitations caused by environmental factors. These factors include the emergence of new SARS-CoV-2 variants, changing COVID-19 infection rates, vaccination rates and number of vaccines administered.

## Conclusions

In unvaccinated elderly individuals (aged ≥ 65 years), the NVX-CoV2373 primary and booster vaccination strategy would be cost-effective compared with no vaccination. Similarly, in elderly individuals who have received a primary vaccination with a COVID-19 mRNA vaccine, an NVX-CoV2373 booster vaccination strategy would be cost-effective compared with no booster vaccination.

## Funding

This study was funded by Takeda Pharmaceutical Company Limited, Tokyo, Japan.

## CRediT authorship contribution statement

**Masafumi Kato:** Conceptualization, Formal analysis, Methodology, Project administration, Validation, Visualization, Writing – original draft, Writing – review & editing, Investigation. **Takayori Ono:** Conceptualization, Formal analysis, Investigation, Methodology, Validation, Visualization, Writing – original draft, Writing – review & editing. **Hisato Deguchi:** Validation, Writing – review & editing, Formal analysis, Investigation, Methodology. **Norio Ohmagari:** Methodology, Writing – review & editing. **Ataru Igarashi:** Methodology, Writing – review & editing.

## Declaration of competing interest

The authors declare the following financial interests/personal relationships which may be considered as potential competing interests: Masafumi Kato reports a relationship with Takeda Pharmaceutical Company Limited that includes: employment. Takayori Ono reports a relationship with Takeda Pharmaceutical Company Limited that includes: employment. Hisato Deguchi reports a relationship with Takeda Pharmaceutical Company Limited that includes: employment. Ataru Igarashi reports a relationship with Takeda Pharmaceutical Company Limited that includes: funding grants and speaking and lecture fees. Ataru Igarashi reports a relationship with Pfizer that includes: consulting or advisory. Ataru Igarashi reports a relationship with Moderna that includes: speaking and lecture fees. Ataru Igarashi reports a relationship with Shionogi that includes: speaking and lecture fees.

## Data Availability

The data that support the findings of this study are available from Medical Data Vision Co., Ltd., but were used under licence for the current study; therefore, restrictions apply, and the data are not publicly available. For inquiries about access to the data set used in this study, please contact Medical Data Vision Co., Ltd. (https://www.mdv.co.jp/; ebm_sales@mdv.co.jp).

## References

[1] WHO COVID-19 Dashboard. Geneva: World Health Organization, 2020. Available online: https://covid19.who.int/ (Accessed 6 September 2023).

[2] Yamamoto N., Takahashi Y., Hayashi S. (2021). Legal and regulatory processes for Japan's COVID-19 immunization program. Vaccine.

[3] Ministry of Health, Labour and Welfare. Infectious disease information: COVID-19 vaccines. Available from: https://www.mhlw.go.jp/stf/covid-19/vaccine.html (Accessed 17 June 2023).

[4] Ministry of Health, Labour and Welfare. Health Sciences Council (Immunization/Vaccine Subcommittee). Available from: https://www.mhlw.go.jp/stf/newpage_31559.html (Accessed 22 March 2023).

[5] Ministry of Health, Labour and Welfare. Health Sciences Council (Immunization/Vaccine Subcommittee). Available from: https://www.mhlw.go.jp/stf/newpage_36489.html (Accessed 12 December 2023).

[6] Takeda. Takeda announces approval of Nuvaxovid® COVID-19 vaccine for primary and booster immunization in Japan. 2022.

[7] Li R., Liu H., Fairley C.K., Zou Z., Xie L., Li X. (2022). Cost-effectiveness analysis of BNT162b2 COVID-19 booster vaccination in the United States. Int J Infect Dis.

[8] Heath P.T., Galiza E.P., Baxter D.N., Boffito M., Browne D., Burns F. (2021). Safety and efficacy of NVX-CoV2373 COVID-19 vaccine. N Engl J Med.

[9] Institute of Tropical Medicine, Nagasaki University. Vaccine effectiveness real-time surveillance for SARS-CoV-2 (VERSUS) study, 7th report. Available from: https://www.tm.nagasaki-u.ac.jp/versus/results/20221221.html (Accessed 14 February 2023).

[10] Chemaitelly H., Tang P., Hasan M.R., AlMukdad S., Yassine H.M., Benslimane F.M. (2021). Waning of BNT162b2 vaccine protection against SARS-CoV-2 infection in Qatar. N Engl J Med.

[11] Goldberg Y., Mandel M., Bar-On Y.M., Bodenheimer O., Freedman L., Haas E.J. (2021). Waning immunity after the BNT162b2 vaccine in Israel. N Engl J Med.

[12] Ministry of Internal Affairs and Communications. Population, demographics and number of households on basic resident registration. Available from: https://www.soumu.go.jp/main_sosiki/jichi_gyousei/daityo/jinkou_jinkoudoutai-setaisuu.html (Accessed 14 February 2023).

[13] Ministry of Health, Labour and Welfare. Visualizing the data: information on COVID-19 infections. Available from: https://covid19.mhlw.go.jp/en/ (Accessed 03 October 2023).

[14] Digital Agency. Open data on COVID-19 vaccination record system (VRS). Available from: https://info.vrs.digital.go.jp/opendata (Accessed 19 January 2023).

[15] National Institute of Infectious Diseases. Preliminary report: estimated incubation period of SARS-CoV-2 variant B.1.1.529 (omicron). Available from: https://www.niid.go.jp/niid/ja/2019-ncov/2551-cepr/10903-b11529-period.html (Accessed 14 February 2023).

[16] National Institute of Infectious Diseases. SARS-CoV-2 variant B.1.1.529 (omicron), 6th report. Available from: https://www.niid.go.jp/niid/ja/2019-ncov/2551-cepr/10900-sars-cov-2-b-1-1-530.html (Accessed 14 February 2023).

[17] Ibaraki Prefecture. COVID-19 vaccination team. Available from: https://www.pref.ibaraki.jp/1saigai/2019-ncov/covid-19_vaccine/team.html#vaccine-sessyurekibetu (Accessed 14 February 2023).

[18] Ministry of Health, Labour and Welfare. The 80th New Coronavirus Infectious Disease Control Advisory Board. Document 5-2: Severity rate and fatality rate in the 6th wave. Available from: https://www.mhlw.go.jp/stf/seisakunitsuite/bunya/0000121431_00333.html (Accessed 14 February 2023).

[19] Koichi Fukunaga, Principal investigator. Health Labour Sciences Research Grant, administrative policy field, Health Labour Sciences Special Research. Fundamental research for understanding the actual status of long-term complications of COVID-19 infection and elucidating pathophysiology. 2022. Available from: https://mhlw-grants.niph.go.jp/project/145956 (Accessed 14 February 2023).

[20] Metry A, Pandor A, Ren S, Shippam A, Clowes M, Dark P, Mcmullan R, Stevenson M. Therapeutics for people with COVID-19 [ID4038]. A multiple technology appraisal. School of Health and Related Research (ScHARR). 2022. Available from: https://www.nice.org.uk/guidance/gid-ta10936/documents/assessment-report (Accessed 14 February 2023). 10.3310/NAFW3527 PMC10591210 37840452

[21] Reddy K.P., Shebl F.M., Foote J.H.A., Harling G., ScottPanella C J.A. (2021). Cost-effectiveness of public health strategies for COVID-19 epidemic control in South Africa: a microsimulation modelling study. Lancet Glob Health..

[22] Ministry of Health, Labour and Welfare. Meeting material of the Subcommittee for Vaccines, Health and Science Council, MHLW. Available from: https://www.mhlw.go.jp/content/10906000/000564407.pdf (Accessed 6 August 2023).

[23] Ibaraki Prefecture. Response to the new corona virus infection. Change in situation 1 (decline in hospitalization rate) [Announced December 1, 2020]. Available from: https://www.pref.ibaraki.jp/1saigai/2019-ncov/221201_teireikaiken.html (Accessed 14 February 2023).

[24] Shiroiwa T., Noto S., Fukuda T. (2021). Japanese population norms of EQ-5D-5L and health utilities index mark 3: disutility catalog by disease and symptom in community settings. Value Health.

[25] Tsuzuki S., Miyazato Y., Terada M., Morioka S., Ohmagari N., Beutels P. (2022). Impact of long-COVID on health-related quality of life in Japanese COVID-19 patients. Health Qual Life Outcomes.

[26] Shiroiwa T., Sung Y.-K., Fukuda T., Lang H.-C., Bae S.-C., Tsutani K. (2010). International survey on willingness-to-pay (WTP) for one additional QALY gained: what is the threshold of cost effectiveness?. Health Econ.

[27] Ministry of Health, Labour and Welfare. Vaccination basic policy subcommittee materials: cost-effectiveness estimation method for vaccination https://www.mhlw.go.jp/file/05-Shingikai-10601000-Daijinkanboukouseikagakuka-Kouseikagakuka/0000207079.pdf (Accessed 28 June 2023).

[28] Ministry of Health, Labour and Welfare. Visualizing the data: information on COVID-19 infections. Available from: https://covid19.mhlw.go.jp/ (Accessed 1 January 2023).

[29] FASCINATE study group. Preliminary report of a case-control study examining the efficacy of COVID-19 vaccines, 4th report: efficacy during the omicron (BA.1/BA.2 and BA.5) epidemic. Available from: https://www.mhlw.go.jp/content/10900000/000977543.pdf (Accessed 14 February 2023).

[30] Statistics Bureau of Japan. Labor force survey, 2022. October 2022. Available from: https://www.e-stat.go.jp/stat-search/files?page=1&layout=datalist&toukei=00200531&tstat=000000110001&cycle=1&year=20220&month=24101210&tclass1=000001040276&tclass2=000001040283&tclass3=000001040284&result_back=1&tclass4val=0 (Accessed 14 February 2023).

[31] Statistics Bureau of Japan. 2021 statistical survey on wage structure. Available from: https://www.e-stat.go.jp/stat-search/files?page=1&layout=datalist&toukei=00450091&tstat=000001011429&cycle=0&tclass1=000001164106&tclass2=000001164107&tclass3=000001164113&tclass4val=0 (Accessed 5 September 2023).

[32] Tomidokoro D., Asai Y., Hayakawa K., Kutsuna S., Terada M., Sugiura W. (2023). Comparison of the clinical characteristics and outcomes of Japanese patients with COVID-19 treated in primary, secondary, and tertiary care facilities. J Infect Chemother.

[33] Tsigaris, Panagiotis and Teixeira da Silva, Jaime A. and Honma, Masayoshi, The Impact of COVID-19 on Japan’s Economic Outlook (February 27, 2023). Available at SSRN: https://ssrn.com/abstract=4372146.

[34] Digital Agency. COVID-19 vaccination status. Available from: https://info.vrs.digital.go.jp/dashboard/ (Accessed 6 September 2023).

[35] Alves K., Plested J.S., Galbiati S., Chau G., Cloney-Clark S., Zhu M. (2023). Immunogenicity and safety of a fourth homologous dose of NVX-CoV2373. Vaccine.

[36] Mallory R.M., Formica N., Pfeiffer S., Wilkinson B., Marcheschi A., Albert G. (2022). Safety and immunogenicity following a homologous booster dose of a SARS-CoV-2 recombinant spike protein vaccine (NVX-CoV2373): a secondary analysis of a randomised, placebo-controlled, phase 2 trial. Lancet Infect Dis.

[37] Kuriyama K., Murakami K., Masuda T., Sugiura K., Sakui S., Schuring R.P. (2023). Immunogenicity and safety of a single booster dose of NVX-CoV2373 (TAK-019) in healthy Japanese adults who had previously received a primary series of COVID-19 mRNA vaccine: primary analysis report of a phase 3 open-label trial. Vaccine.

[38] Di Fusco M., Marczell K., Deger K.A., Moran M.M., Wiemken T.L., Cane A. (2022). Public health impact of the Pfizer-BioNTech COVID-19 vaccine (BNT162b2) in the first year of rollout in the United States. J Med Econ.

[39] Fernandes R.R.A., Santos M.S., Magliano C.A.S., Tura B.R., Macedo L.S.D.N., Padila M.P. (2022). Cost utility of vaccination against COVID-19 in Brazil. Value Health Reg Issues.

[40] Kohli M., Maschio M., Becker D., Weinstein M.C. (2021). The potential public health and economic value of a hypothetical COVID-19 vaccine in the United States: use of cost-effectiveness modeling to inform vaccination prioritization. Vaccine.

[41] Orlewska K., Wierzba W., Śliwczynski A. (2022). Cost-effectiveness analysis of COVID-19 vaccination in poland. Arch Med Sci.

[42] Xiong X., Li J., Huang B., Tam T., Hong Y., Chong K.-C. (2022). Economic value of vaccines to address the COVID-19 pandemic in Hong Kong: a cost-effectiveness analysis. Vaccines.

[43] Medical Fee Points Table 2022 April. Tokyo: Igakutsushinsha Co.; 2022.

